# Wear Characteristics of Mg Alloy AZ91 Reinforced with Oriented Short Carbon Fibers

**DOI:** 10.3390/ma15144841

**Published:** 2022-07-12

**Authors:** Sabbah Ataya, Mohamed M. El-Sayed Seleman, Fahamsyah H. Latief, Mohamed M. Z. Ahmed, Khalil Hajlaoui, Ahmed M. Soliman, Naser A. Alsaleh, Mohamed I. A. Habba

**Affiliations:** 1 Department of Mechanical Engineering, College of Engineering, Imam Mohammad Ibn Saud Islamic University, Riyadh 11432, Saudi Arabia; smataya@imamu.edu.sa (S.A.); fhlatief@imamu.edu.sa (F.H.L.); kmhajlaoui@imamu.edu.sa (K.H.); naalsaleh@imamu.edu.sa (N.A.A.); 2 Department of Metallurgical and Materials Engineering, Faculty of Petroleum and Mining Engineering, Suez University, Suez 43512, Egypt; mohamed.elnagar@suezuniv.edu.eg; 3 Department of Mechanical Engineering, Faculty of Engineering and Science, Universitas Nasional, Jakarta 12520, Indonesia; 4 Mechanical Engineering Department, College of Engineering at Al Kharj, Prince Sattam bin Abdulaziz University, Al Kharj 16273, Saudi Arabia; 5 Department of Mechanical Engineering, College of Engineering, Jouf University, Sakaka 72388, Saudi Arabia; amsoliman@ju.edu.sa; 6 Mechanical Department, Faculty of Technology & Education, Suez University, Suez 43518, Egypt; mohamed.atia@suezuniv.edu.eg

**Keywords:** magnesium alloy AZ91, short carbon fibers, microstructure, compressive strength, wear resistance, wear mechanisms, wear debris

## Abstract

Light-weight metal matrix composites, especially magnesium-based composites, have recently become more widespread for high-efficiency applications, including aerospace, automobile, defense, and telecommunication industries. The squeeze cast AZ91 base material (AZ91-BM) and its composites having 23 vol.% short carbon fibers were fabricated and investigated. The composite specimens were machined normal to the reinforced plane (Composite-N) and parallel to the reinforced plane (Composite-P). All the as-casted materials were subjected to different tests, such as hardness, compression, and wear testing, evaluating the mechanical properties. Dry wear tests were performed using a pin-on-disk machine at room temperature under different applied wear loads (1–5 N) and different sliding distances ($0.4461\times 10^{4}$–$3.12\times 10^{4}$ m). The microstructures and worn surfaces of the fabricated AZ91-BM and the two composite specimens were investigated using a scanning electron microscope (SEM) equipped with an energy dispersive spectroscopy (EDS) advanced analysis system. The wear debris was collected and investigated also under the SEM. The results showed significant improvement in hardness, compressive strength, and wear resistance of the composite specimens (Composite-N and Composite-P) over the AZ91-BM. The compressive strength and wear resistance are more fibers orientation sensitive than the hardness results. When the fiber orientation is parallel to the sliding direction (Composite-N), the weight loss is somewhat lower than that of the fiber orientation perpendicular to the sliding direction (Composite-P) at a constant wear load of 2 N and the sliding distances of $0.4461\times 10^{4}$, $1.34\times 10^{4\;},$ and $2.23\times 10^{4}$ m. In contrast, the weight loss of Composite-P is lower than Composite-N, especially at the highest sliding distance of $3.12\times 10^{4}$ m due to the continuous feeding of graphite lubricant film and the higher compressive strength. Plastic deformation, oxidation, and abrasive wear are the dominant wear mechanisms of AZ91-BM; in contrast, abrasive and delamination wear are mainly the wear mechanisms of the two composites under the applied testing conditions.

## 1. Introduction

Nowadays, the demand for materials weight reduction for structural applications has significantly increased. For this purpose, magnesium (Mg) alloys are promising candidates to be introduced since they have outstanding physical and chemical properties, which are considered to have expectant prospects in the areas of aerospace, automotive, and electronics [1]. On the other hand, Mg alloys have low strength and poor wear resistance, which limits their applications for structural and anti-friction applications [2]. It is well known that Mg-based composites are superior in certain aspects compared with the monolithic magnesium alloy, such as high specific strength and excellent damping capacity, which have become one of the research focuses of many engineering applications.

Many scientists presented that the stability and mechanical properties of Mg alloys at high temperatures can be increased by adding the reinforcements into Mg alloys as a matrix to make Mg-based composites have better performance compared with its alloys [3,4]. Lately, many researchers put their concern on using discontinuous particles as reinforcement for metal-matrix composite (MMC) since they are low-cost and easy to fabricate through secondary processing, have better mechanical properties, and have good homogeneity [5,6]. Noted that one of the requirements for structural applications, the materials should have excellent wear resistance during services. El-Sayed Seleman et al. [7] reported that the incorporation of graphite powders up to 20 wt.% in the AA6016 aluminum matrix improved the wear resistance of aluminum/graphite composites compared to AA6016 aluminum alloy.

Liu et al. [8] stated that short carbon fiber lowered direct contact between the aluminum matrix and counterpart and enhanced the wear resistance. This composite system has been produced by the vacuum pressure infiltration technique.

Furthermore, the mechanical and wear behavior of boron carbide and graphite-reinforced AZ91 magnesium matrix hybrid composite was discussed by Aatthisugan et al. [9]. As their results, the incorporation of boron carbide into AZ91 alloy enhanced the wear behavior of Mg composite, whereas the addition of graphite into AZ91–B_4_C composite as a hybrid reinforcement reduced the wear resistance of Mg composite. Lim et al. [10] investigated the sliding wear behavior of AZ91/SiCp composites at various loads ranging from 10−30 N. Compared with its matrix alloy, the wear behavior of Mg composite at 10 N was significantly improved by a 15–30% increment. A study on the dry sliding wear behavior of globular AZ91 alloy and AZ91/SiCp composites was carried out by Garcia-Rodriguez et al. [11]; they summarized that the wear mechanism of globular AZ91 alloy was varied at different loads and sliding speeds. An investigation on the wear resistance of AE42-based composite reinforced with 23% vol.% carbon short fibers was conducted by Ataya et al. [12], and they confirmed that the use of various loads at a constant sliding distance or vice versa promoted more weight loss of the AE42 matrix than its composite.

Herein, the carbon short fibers are incorporated in the AZ91 alloy to produce an Mg-based composite by the squeeze casting technique. To the best of our knowledge, there is still little published work found on the field of wear behavior of AZ91 alloy reinforced with oriented high volume fraction short carbon fibers. In fact, carbon short fibers are considered an important material for different engineering applications when used as reinforcement into Mg matrix to produce composites due to their isotropic and stable mechanical performance [13]. Thus, the combination of the AZ91 alloy and the short carbon fibers is interesting to be investigated to explore the wear behavior of the Mg-based composite containing an oriented high-volume fraction. Therefore, the present work aims to comprehend the wear behavior of the AZ91/23% vol.% short carbon fiber composite materials for the specimens machined normal and parallel to the reinforcing plane. The influence of different loads and sliding distances of the as-cast AZ91 base material (BM) and the produced composites were examined in the light of the oriented short carbon fiber. The microstructures of the produced materials are investigated; in addition, an extensive study of the worn surface under SEM is also undertaken to show the role of the reinforcing carbon fibers in the wear process. The hardness and compressive strength of the produced materials are also evaluated.

## 2. Methodology

### Materials

**Figure 1:**
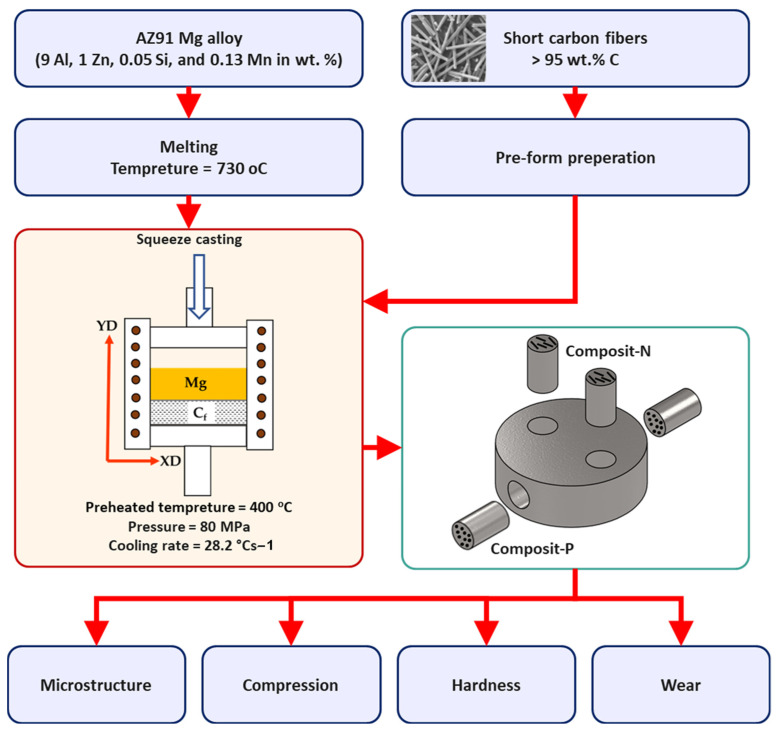
The flow chart summarizes the experimental procedure to produce and characterize the AZ91-BM and its composites.

The starting materials to produce Mg-based composite containing a high-volume fraction of short carbon fibers via an advanced squeeze casting technique were AZ91 and short carbon fibers. The used carbon fibers were coated with silicon to improve the wettability with the AZ91 matrix and to hinder carbide formation at the interface between the matrix and the reinforcement. Researchers reported using many coating materials such as pyrolytic carbon layer [14], copper and nickel [15], alumina [16], and silicon carbide [17]. The carbon fibers were supplied by SIGRAFIL (SGL Carbon GmbH), Germany. According to the supplier, the diameter and length were 5–6 µm and 80–120 µm, respectively, and the physicomechanical properties were 1.76 g/cm^3^ density and 280 GPa elastic modulus. The composite was designed to have 23 vol.% short carbon fibers. Figure 1 summarizes the experimental procedure to produce and characterize the AZ91-BM and its composites. The as-cast-produced composite was machined normally and parallel to the reinforcing plane, as sketched in the flow chart (Figure 1). In the current study, the composite specimen machined normal to the reinforced plane is named Composite-N, and that machined parallel to the reinforced plane is named Composite-P.

**Figure 2:**
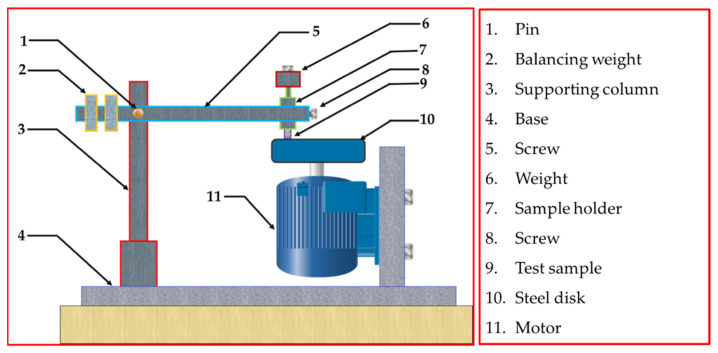
Schematic of homemade pin-on-disk wear testing machine.

Cylindrical specimens of the AZ91-BM, Composite-N, and Composite-P were machined in the dimensions of 6 mm in diameter and 9 mm in length for further testing and investigations. Hardness was measured using a Vickers hardness testing machine (Model-HWDV-7S) at 2 N load and 15 s dwell time loading conditions. Compression testing was applied at room temperature using a Universal Testing Machine (Schenck-Trebel RMC100, Deer Park, NY, USA) with a cross head speed of 0.01 mm/min. Microstructures were examined for the AZ91-BM and both composite specimens using a scanning electron microscope, Quanta FEG 250, Hillsboro, OR, USA, equipped with an energy-dispersive X-ray spectrometry (EDS) advanced system. Two SEM detectors, low k- Volt High-Contract Detector (vCD) and Everhart–Thornley Detector (ETD), were used to distinguish the microstructure features. Wear tests using a homemade pin-on-disc machine (WT-M1-SSMMR-CSE, Suez University, Suez, Egypt, Figure 2) were used to evaluate the wear behavior of the AZ91-BM and AZ91/23 vol.% short carbon fibers composites. The tested specimens were rubbed against a hard steel disk has a hardness of 64 HRC at different loads and sliding distances. The applied wear load ranged from 1 to 5 N, and the sliding distance ranged from $0.4461\times 10^{4}$ to $3.12\times 10^{4}$ m. After testing, the debris was collected for each wear condition. The worn surfaces and the collected debris were investigated using the SEM-EDS system.

## 3. Results and Discussion

### 3.1. Microstructures AZ91-BM and Composite Specimens

**Figure 3:**
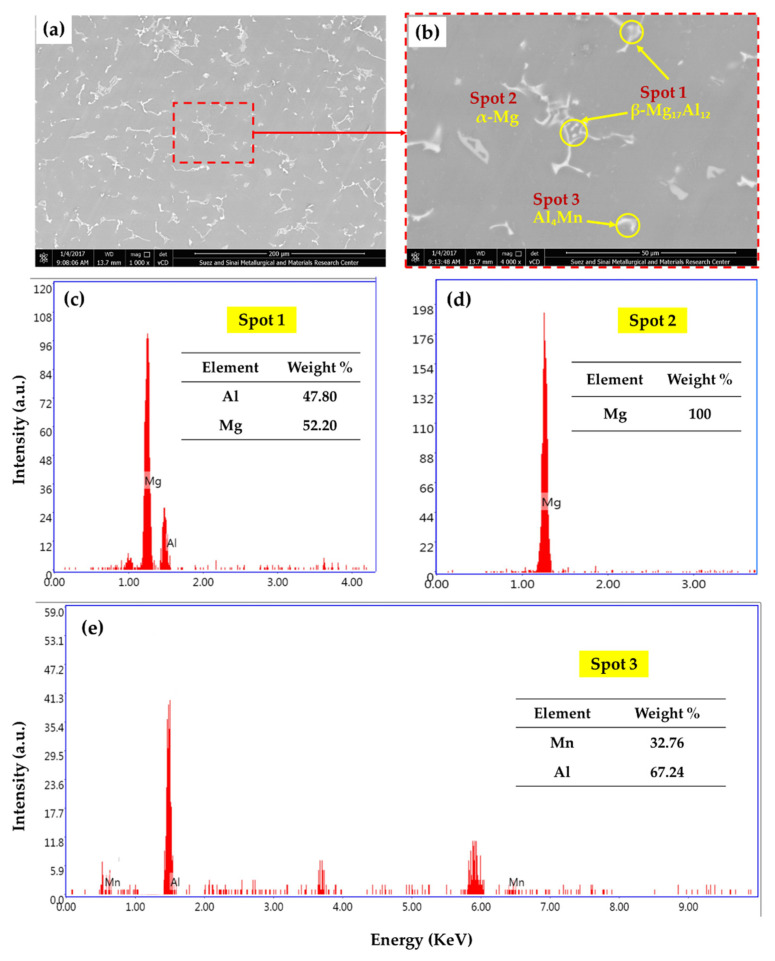
SEM images at (**a**) low and (**b**) high magnification of the AZ91-BM and the EDS analysis of the AZ91 intermetallics; (**c**) spot 1, (**d**) spot 2, and (**e**) spot 3 in subfigure (**b**).

The morphologies of the intermetallic phases in Mg alloys are governed by some parameters, including the applied casting process and its variables, alloying elements, and cooling rate [18,19,20]. It is worth mentioning that the physical and mechanical properties of the as-cast material are related directly to the microstructure features. Thus, the SEM-EDS examinations were conducted to investigate the microstructure features of the as-cast AZ91-BM and the produced composites. Figure 3 shows SEM images of the AZ91-BM microstructure and the formed intermetallics. The microstructure involves a large α-Mg (Spot 2) dendritic structure (gray areas) with secondary arms, as given in Figure 3a,b. Two intermetallics were detected in the microstructure of AZ91-BM. The first appears as bright layers at the α-Mg grain boundaries (eutectic phase of β-Mg_17_Al_12_ intermetallic and α-Mg lamellar). It also appeared as a solid bright area on the α-Mg grain boundaries (spot 1 in Figure 3b and represented in Figure 3c). The different shapes of the β-Mg_17_Al_12_ intermetallics are attributed to the non-equilibrium solidification [21]. The second intermetallic is Al_4_Mn (spot 3 in Figure 3b and represented in Figure 3e). These microstructure features in terms of intermetallic phases, morphologies, and their dispersion of the as-cast AZ91 matrix are typically agreed well with that examined and reported for the same alloy material in other works [22,23,24].

**Figure 4:**
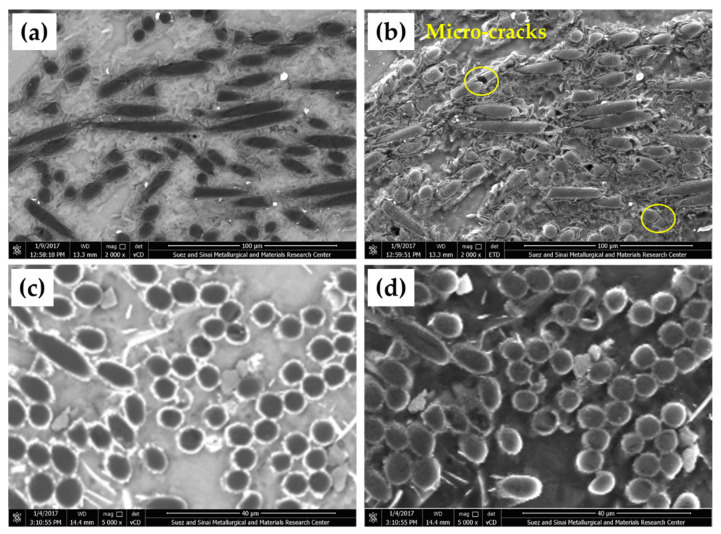
SEM images show the microstructures of Composite-N, (**a**) VCD, (**b**) EDT and Composite-P, (**c**) VCD, and (**d**) EDT.

Figure 4 shows the microstructures of the AZ91/23 vol.% short carbon fibers detected in Composite-N and Composite-P specimens. The carbon fiber dispersed and bonded well with the AZ91 matrix in both composite specimens. In addition, localized agglomeration of carbon fibers is also remarked in the microstructure investigation, as the carbon fibers tend to agglomerate [6] and as the result of the used pressure during the squeeze casting process. In composite-N, the carbon fibers mostly appeared in the longitudinal direction (Figure 4a,b). Some fibers are micro-cracked in the transverse direction with keeping their position in the Mg matrix, as shown in Figure 4b (ETD mode). This fiber fracture may be ascribed to the difference in the thermal expansion coefficient of the AZ91 Mg matrix and carbon fibers. In addition, interfacial debonding and sliding may also be expected in the Mg/carbon fiber composite system [25]. The axial thermal expansion coefficient of the carbon fibers has a more pronounced effect than that of the transverse direction on the interfacial bonding between the fibers and the Mg matrix composite during fast cooling of molten Mg to room temperature. This leads to partial debonding at the interface between the Mg matrix and the longitudinal fiber direction instead of the cross-section. In Composite-P, the circular cross-sections of the carbon fibers (Figure 4c,d) lie in the range of the as-received short carbon fiber diameters (5–6 µm). It can be remarked from Figure 4d (ETD mode) that the carbon fibers are bonded well with the Mg matrix without any cracking in the carbon cross-sections.

### 3.2. Mechanical Properties

The AZ91-BM hardness value is 70 ± 3 HV. This value increased for the composite specimens containing 23 vol.% short carbon fibers to be 106 ± 4 HV and 111 ± 2 HV for the Composite-N and Composite-P, respectively. This improvement in hardness is ascribed to the action of adding a high-volume fraction (23 vol.%) of reinforcing carbon fiber. This result agreed well with that reported by many authors for different Mg-based composites containing different volume fractions of carbon fibers [12,22,26].

**Figure 5:**
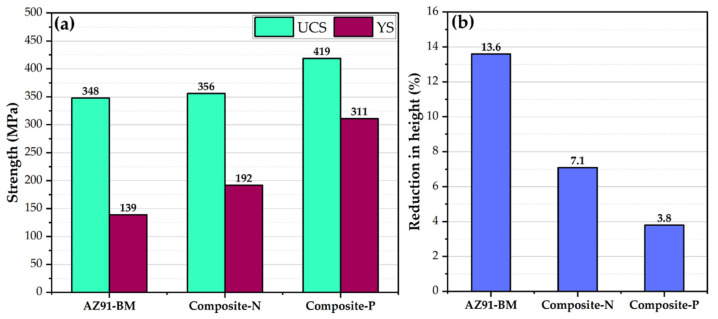
(**a**) Ultimate compressive strength and yield strength and (**b**) reduction in the height of the compression tested AZ91 matrix alloy and the composites.

The compressive properties of the AZ91-BM and the two composites are presented in Figure 5, where Figure 5a illustrates the yield compressive strength (YCS) and ultimate compressive strength (UCS), and Figure 5b shows the influence of 23 vol.% short carbon fibers on the ductility of the compression tested specimens in terms of reduction in height (R%). It can be remarked that the two composites possess higher YCS and UCS than AZ91-BM (Figure 5a). The UYS of the Composite-N specimens improved by 38% over AZ91-BM, whereas no significant improvement in the UCS of the Composite-N compared to BM. For Composite-P, the enhancement in both YCS and UCS is remarkably observed over AZ91-BM and attained the improvement percentage of 124 and 20, respectively. This improvement in strength for the composite specimens can be attributed to the high modulus of elasticity of the short carbon fibers compared to the AZ91-BM [27,28]. The ductility loss for Composite-N and Composite-P are 48 and 72%, respectively, compared to AZ91-BM. The increase in hardness and strength is usually at the expense of ductility loss.

**Figure 6:**
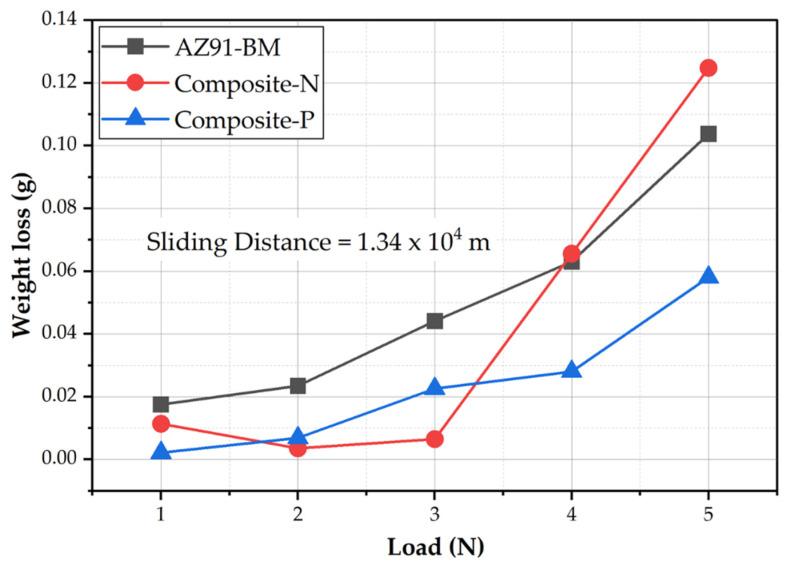
Weight loss against the applied wear loads for AZ91-BM and composite specimens wear-tested at a constant sliding distance of 1.34 × 10^4^ m.

AZ91-Mg alloy is widely used in automotive and aerospace industries due to its excellent combination of low density and high thermal conductivity. However, it suffers from poor wear resistance. To overcome this, high specific strength reinforcement phases (fibers, particulates, and whiskers) have been recommended to introduce to the Mg matrix to improve its wear resistance via producing AZ91-based composites [12,14]. Moreover, the wear behavior of the fiber-reinforced composite materials is governed by hardness, compressive strength, and fiber orientation with respect to sliding direction. The influence of oriented fibers on the wear properties is more complicated (especially for discontinuous fiber-reinforced composites) because of the random distribution of fibers. Thus, it is important to explore the wear properties of AZ91-BM and the produced composite specimens having oriented 23 vol.% short carbon fibers. The first group of the wear-tested specimens was conducted at a constant sliding distance of $1.34\times 10^{4\;}$ m and different loads of 1, 2, 3, 4, and 5 N for the AZ91-BM, Composite-N, and Composite-P. Figure 6 shows the weight loss of the wear-tested materials (AZ91-BM and its composite specimens) against the applied wear loads. It can be observed that the weight loss of all the wear-tested materials increases with increasing the wear loads from 1 to 5 N for the BM and the two composite specimens. The weight loss of AZ91-BM is higher than that of both composites for all the applied wear loads from 1 to 3 N. After that, the Composite-N shows the highest weight loss at 5 N, whereas Composite-P displays the lowest weight loss at the higher wear loads of 4 and 5 N. The higher weight loss of AZ91-BM compared to the two composite specimens at a wide range of the applied wear loads (1–3 N) is ascribed to its lower hardness and compressive strength values compared to the AZ91-based composite specimens reinforced with the short carbon fibers. Carbon fibers have graphite-like layers, which are weakly bonded together. These layers have a very low coefficient of friction while sliding on another surface. During the wear testing, carbon fibers are fractured and spread on the worn surface of composites, forming a solid lubricant film. This lubricating film reduces the friction and dissipates the generated heat between the two rubbing surfaces of the wear-tested composite specimens and the steel disk counterpart. This leads to a reduced weight loss of both composites (Figure 6). Furthermore, at the mild wear loads of 2 and 3 N, the Composite-N shows an improvement in wear properties compared to the other tested specimens (AZ91-BM and Composite-P) because of the largest lubricant feeding area as the carbon fibers parallel to the hard rubbing surface. This promotes more graphite film than in the case of the fiber normal to the hard rubbing surface (Composite-P). At the same time, the applied shear force on the worn surface is lower than the cohesion force between the carbon fibers and the AZ91 matrix, which makes the carbon fibers achieve their role of lubrication. In contrast, at the wear loads of 4 and 5 N, the applied shear force on the worn surface is higher than the cohesion force between the carbon fibers and the AZ91 matrix. This causes carbon fibers to pull out due to the presence of the interfacial deboning as a result of thermal expansion mismatch between carbon fibers and the AZ91 matrix. The thermal expansion coefficient of AZ91 alloy is 26.8 × 10^−6^ K^−1^, while the axial thermal expansion coefficient of carbon fiber is 1.48 × 10^−6^ K^−1^ [25]. The pull-out of carbon fibers causes material loss without utilizing the inherent lubricity property. This phenomenon is remarked only with the Composite-N and combined with delamination layers of AZ91 matrix alloy due to the accumulated friction heat and severe plastic deformation. These cause deterioration in the wear properties of Composite-N compared to the AZ91-BM, and the Composite-P at the highest applied wear load of 5 N.

**Figure 7:**
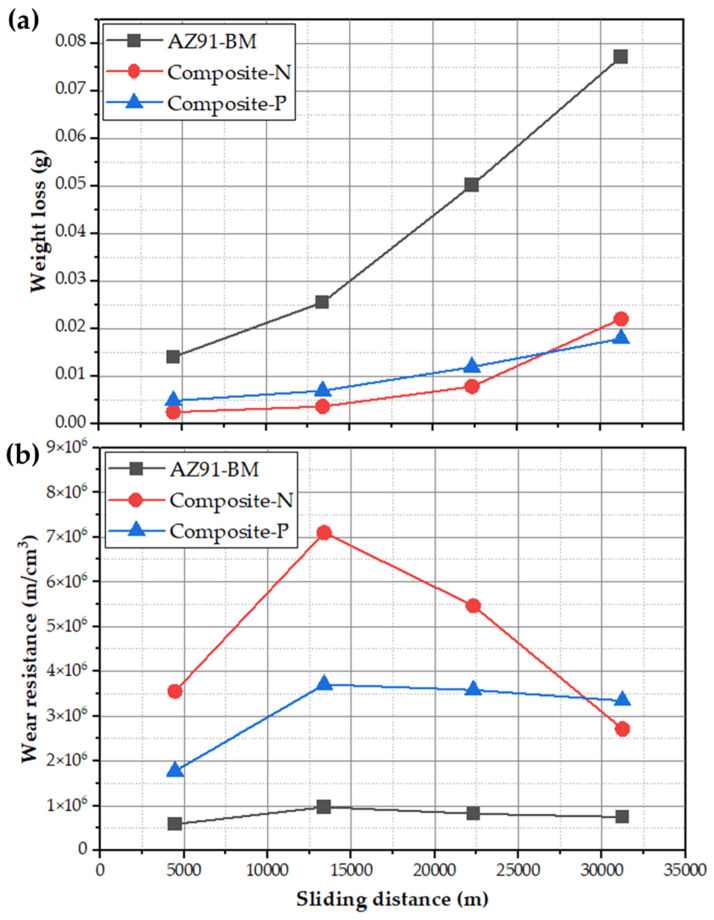
(**a**) Weight loss and (**b**) wear resistance against the applied sliding distances for AZ91-BM and composite specimens wear-tested at a constant wear load of 2 N.

In order to examine the wear behavior in terms of weight loss of the AZ91-BM, Composite-N, and Composite-P at different sliding distances, the second group of the wear-tested specimens was conducted under a constant mild load of 2 N at different running sliding distances of $0.4461\times 10^{4\;}$, $1.34\times 10^{4\;}$, $2.23\times 10^{4\;}$ and $3.12\times 10^{4\;}$ m, as given in Figure 7a. It can be seen that both composites (Composite-N and Composite-P) display notable lower weight loss than AZ91-BM, indicating significant improvement in wear resistance in the presence of 23 vol.% oriented short carbon fibers. It is also observed that the weight loss values of Composite-N are lower than that given by Composite-p and AZ91-BM at the sliding distance range from $0.4461\times 10^{4\;}$ to $2.23\times 10^{4\;}$ m, whereas, at the highest sliding distance of $3.12\times 10^{4\;}$ m, Composite-P shows the lowest weight loss of 0.01785 g compared to the AZ91-BM (0.0771 g) and the Composite-N (0.022 g). At a mild wear load of 2 N and applying the highest sliding distance of $3.13\times 10^{4\;}$ m, the formed lubricating film is not enough to dissipate the generated friction heat, and then the accumulated heat weakens the bond strength between the carbon fibers and the surrounded Mg matrix as a result of the thermal expansion mismatch between the carbon fibers and the Mg matrix. This leads to the pull-out of the carbon fibers without complete utilizing of its inherent lubrication effect and increases the weight loss of composite-N. In contrast, Composite-P does not suffer from the pull-out fiber phenomenon and utilizes completely the action of graphite fibers in the lubrication. In addition, the contentious lubricant film feeding when the fibers normal to the rotating wear machine desk, the Composite-P keeps its improvement in compressive strength.

Based on the collected data from the wear testing parameters and the density of the wear-tested specimens (AZ91-BM, Composite -N, and Composite-P), the wear properties are plotted as a wear resistance versus the applied sliding distances at a constant load of 2 N (Figure 7b). It can be seen that the incorporation of the oriented 23 vol.% short carbon fibers in the AZ91 matrix improves the wear resistance of the produced composite specimens over the AZ91-BM at all the applied sliding distances. In the beginning, there is an increase in the wear resistance in varying proportions for AZ91-BM, Composite-N, and Composite-P at the sliding distances from $0.4461\times 10^{4}$ to $1.34\times 10^{4\;}$ m. The slight increase in the wear resistance of the AZ91-BM may be ascribed to strain hardening, whereas a significant increase in the wear resistance of Composite-N and Composite-P is remarked at the same sliding distance range. This enhancement is likely due to the addition of a high-volume fraction of short carbon fibers. Above the sliding distance of $1.34\times 10^{4\;}$ m, the wear resistance of Composite-N displays remarked to decrease with increasing the sliding distance. For AZ91-BM and Composite-P, the wear resistance curves show nearly a steady-state behavior with the increase in the sliding distance. Furthermore, Composite-P shows the highest wear resistance of $3.34\times 10^{6}$ m/cm^3^ compared to the wear resistance of AZ91-BM ($0.749\times 10^{6}$ m/cm^3^) and Composite-N ($2.71\times 10^{6}$ m/cm^3^) at the highest sliding distance of $3.12\times 10^{4}$ m (Figure 7b). Finally, the Composite-N shows the lowest weight loss compared to the Composite-P and the AZ91-BM at a constant sliding distance of 1.34 × 10^4^ m and the wear loads of 2 and 3 N. Additionally, it shows the lowest weight loss (the highest wear resistance) compared to the Composite-P and the AZ91-BM at a constant mild wear load of 2 N and the sliding distance range from 0.4461 × 10^4^ to 3.12 × 10^4^ m. In contrast, the Composite-P shows lower weight loss compared to the Composite-N and the AZ91-BM at a constant sliding distance of 1.34 × 10^4^ m and the wear loads of 4 and 5 N. In addition, it shows the lowest weight loss (the highest wear resistance) compared to all the wear-tested specimens, especially at a wear load of 2 N and the highest sliding distance of 3.12 × 10^4^ m.

**Figure 8:**
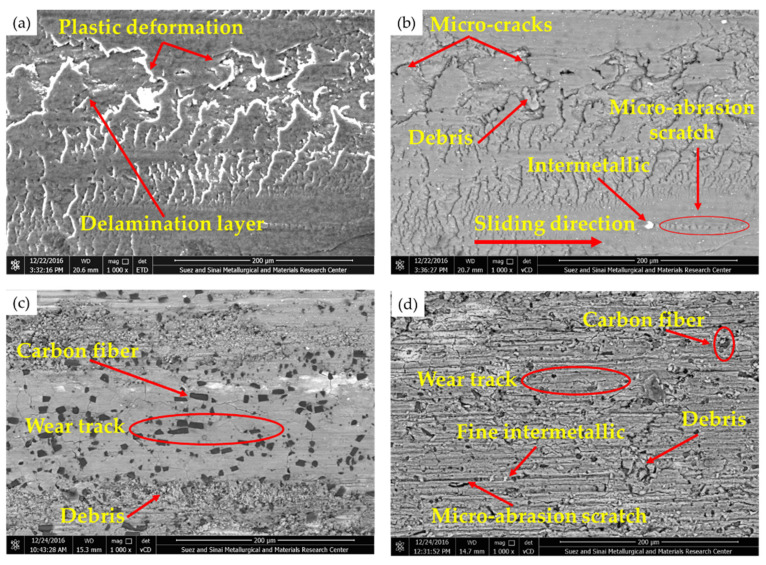
SEM images of the worn surfaces of the wear-tested specimens, where (**a**,**b**) AZ91-BM in ETD and vCD modes, respectively, (**c**) Composite-N and (**d**) Composite-P at the wear test conditions of wear load of 2 N and sliding distance of $1.34\times 10^{4\;}$ m.

**Figure 9:**
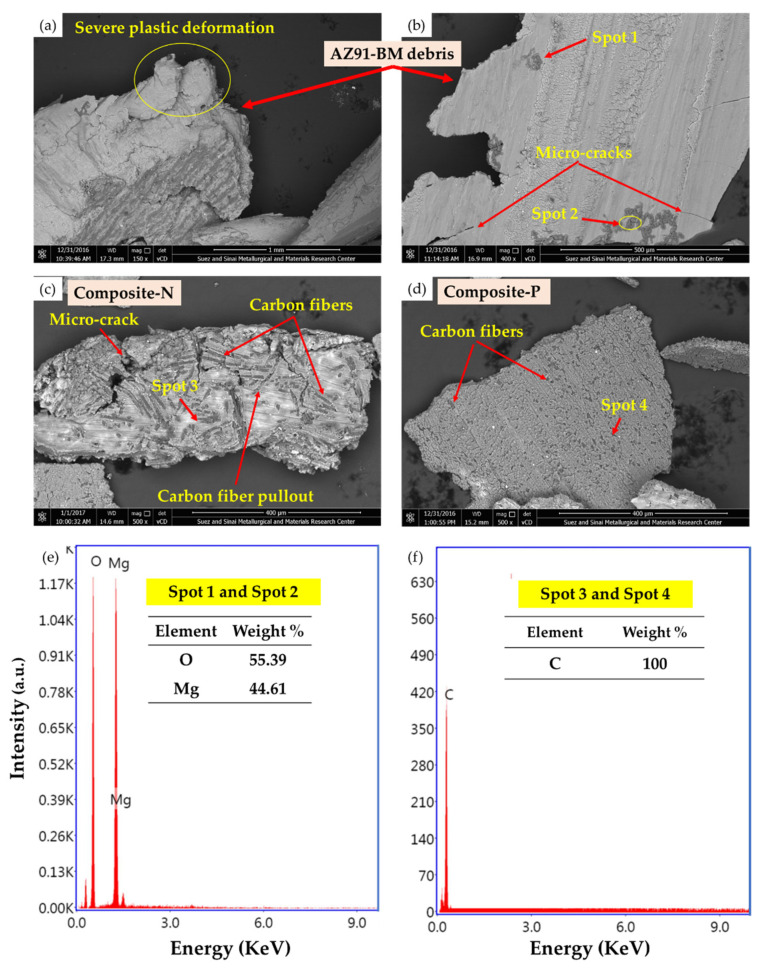
SEM images of the debris of the wear-tested specimens, where (**a**,**b**) AZ91-BM, (**c**) Composite-N (**d**) Composite-P, (**e**) EDS spot analysis of MgO, and (**f**) EDS spot analysis of Carbon fiber.

To understand the wear mechanisms of AZ91-BM and the produced composite specimens containing oriented high-volume fraction of short carbon fibers, the worn surfaces and gathered debris of the wear-tested specimens were examined by SEM. Figure 8 displays the worn surface features of the wear-tested specimens of AZ91-BM, Composite-N, and Composite-P, and Figure 9 illustrates SEM images and EDS analysis of the gathered debris of the AZ91 matrix alloy and the composite specimens. The worn surface of the AZ91-BM (investigated using two SEM detectors, ETD and vCD modes) displays clear damage in the form of continuous scratches parallel to the sliding direction, plastic deformation, transverse microcracks, and delamination layers (Figure 8). There are also small smooth regions interspersed with wear scars. A notable feature in the SEM micro-images is inclined shear plates due to high surface stress [12]. The wear mechanisms are plastic deformation, delamination layer, and abrasion wear. The abrasion wear was caused by the free pull-out intermetallic particles and the formed magnesium oxide (Figure 9e) between the two rubbing surfaces, AZ91-BM and the hard steel disk. During the wear tests, the AZ91-BM is easily scraped due to its lower strength and hardness, which leads to material loss (Figure 9a,b). At the similar wear test condition, the worn surface of the two composites (Composite-N, Figure 8c; and Composite-P, Figure 8d) having 23 vol.% short carbon fibers displays other features. The scratches (wear track) of both composites become shallower than that in the AZ91-BM as a result of the short carbon fibers addition. Furthermore, the inclined Mg shear plates nearly disappeared from the worn surface of the composite specimens. In addition, smearing the worn surface with carbon film is generally remarked (Figure 8c,d). This carbon film lubricates the worn surface of the composites and decreases the degree of delamination [29] and the influence of abrasive wear [12]. This action generally reduces the wear loss of both composites as appears in their size of debris as given in Figure 9c for Composite-N and Figure 9d for Composite-P compared to the AZ91-matrix material’s debris. It is worth mentioning that the AZ91 debris is very large and highly deformed with irregular shapes and dimensions, as shown in Figure 9a,b. The formation of this type of debris can be ascribed to the delamination layers and an abrasive wear effect. However, the wear debris becomes smaller and lightly deformed for AZ91/23 vol.% carbon short fibers the Composite-N and the Composite-P as shown in Figure 9c,d, respectively. This decrease in the size of the debris is also detected in another work [7] and is mainly due to the role of carbon fibers in reducing the probabilities of direct contact between two worn surfaces and dissipating the frictional heat, which finally decreases the severity of micro-cutting effects. The mainly working wear mechanisms of the composites are likely to be abrasive and delamination wear [14]. It is also remarked that the worn surface of the Composite-N is smoother than that of the Composite-P. This may be ascribed to the intensity of the supplied lubricant film in the case of short carbon fibers being parallel to the sliding surface plane. The feeding of graphite film is discontinuous as the graphite fibers are separated by the Mg matrix. In the case of the short carbon fibers being perpendicular to the sliding surface plane, the feeding of graphite film is continuous. This leads to the higher wear resistance of Composite-P than Composite-N, especially at the highest distance of $3.12\times 10^{4}$ m using an applied wear load of 2 N (Figure 7b).

## 4. Conclusions

Based on the obtained results, the following conclusions can be outlined:

1. The reinforcing of AZ91 Mg alloy by 23 vol.% short carbon fibers improved the hardness of the two composites (Composite-N and Composite-P) by not less than 51%.
2. The YCS of the Composite-N and Composite-P were enhanced over AZ91-BM by 38% and 124%, respectively. In addition, Composite-P recorded the highest UCS compared to Composite-N and AZ91-BM.
3. The two composites display notable lower weight loss than AZ91-BM at a constant sliding distance of $1.34\times 10^{4}$ m, and the applied wear loads from 1 to 3 N, indicating significant improvement in wear resistance in the presence of 23 vol.% oriented short carbon fibers.
4. Composite-P shows the lowest weight loss of 0.01785 g compared to the AZ91-BM (0.0771 g) and the Composite-N (0.022 g) at the wear conditions of 2 N applied load and the highest sliding distance of $3.12\times 10^{4\;}$ m.
5. The two composites show higher wear resistance than AZ91-BM at a constant applied wear load of 2 N and various sliding distances from $0.4461\times 10^{4}\;\mathrm{to}\;3.12\times 10^{4}$ m. Furthermore, Composite-P possesses the highest wear resistance at a constant applied wear load of 2 N and the highest sliding distance of $3.12\times 10^{4}$ m.
6. Plastic deformation, oxidation, and abrasive wear are the dominant wear mechanisms of AZ91-BM; in contrast, abrasive and delamination wear are mainly the wear mechanisms of the two composites under the applied testing conditions.

## Data Availability

The data presented in this study are available on request from the corresponding author. The data are not publicly available due to the extremely large size.

## References

[1] Luo K., Zhang L., Wu G., Liu W., Ding W. (2019). Effect of Y and Gd Content on the Microstructure and Mechanical Properties of Mg–Y–RE Alloys. J. Magnes. Alloy.

[2] Lyu J., Kim J., Liao H., She J., Song J., Peng J., Pan F., Jiang B. (2020). Effect of Substitution of Zn with Ni on Microstructure Evolution and Mechanical Properties of LPSO Dominant Mg–Y–Zn Alloys. Mater. Sci. Eng. A.

[3] Tokunaga T., Ohno M., Matsuura K. (2018). Coatings on Mg Alloys and Their Mechanical Properties: A Review. J. Mater. Sci. Technol..

[4] Zhang L., Wang Q., Liao W., Guo W., Li W., Jiang H., Ding W. (2017). Microstructure and Mechanical Properties of the Carbon Nanotubes Reinforced AZ91D Magnesium Matrix Composites Processed by Cyclic Extrusion and Compression. Mater. Sci. Eng. A.

[5] Bakkar A., Ahmed M.M.Z., Alsaleh N.A., Seleman M.M.E.S., Ataya S. (2019). Microstructure, Wear, and Corrosion Characterization of High TiC Content Inconel 625 Matrix Composites. J. Mater. Res. Technol..

[6] Shirvanimoghaddam K., Hamim S.U., Karbalaei Akbari M., Fakhrhoseini S.M., Khayyam H., Pakseresht A.H., Ghasali E., Zabet M., Munir K.S., Jia S. (2017). Carbon Fiber Reinforced Metal Matrix Composites: Fabrication Processes and Properties. Compos. Part A Appl. Sci. Manuf..

[7] El-Sayed Seleman M.M., Ahmed M.M.Z., Ataya S. (2018). Microstructure and Mechanical Properties of Hot Extruded 6016 Aluminum Alloy/Graphite Composites. J. Mater. Sci. Technol..

[8] Liu L., Li W., Tang Y., Shen B., Hu W. (2009). Friction and Wear Properties of Short Carbon Fiber Reinforced Aluminum Matrix Composites. Wear.

[9] Aatthisugan I., Razal Rose A., Selwyn Jebadurai D. (2017). Mechanical and Wear Behaviour of AZ91D Magnesium Matrix Hybrid Composite Reinforced with Boron Carbide and Graphite. J. Magnes. Alloy.

[10] Lim C.Y.H., Lim S.C., Gupta M. (2003). Wear Behaviour of SiCp-Reinforced Magnesium Matrix Composites. Wear.

[11] García-Rodríguez S., Torres B., Maroto A., López A.J., Otero E., Rams J. (2017). Dry Sliding Wear Behavior of Globular AZ91 Magnesium Alloy and AZ91/SiCp Composites. Wear.

[12] Ataya S., Naser Alsaleh B.A., Mohamed El-Sayed Seleman B.M. (2019). Strength and Wear Behavior of Mg Alloy AE42 Reinforced with Carbon Short Fibers. Acta Metall. Sin. (Engl. Lett.).

[13] Yang F., Zhang X., Han J., Du S. (2008). Mechanical Properties of Short Carbon Fiber Reinforced ZrB2-SiC Ceramic Matrix Composites. Mater. Lett..

[14] Qi L., Guan J., Liu J., Zhou J., Wei X. (2013). Wear Behaviors of Cf/Mg Composites Fabricated by Extrusion Directly Following Vacuum Pressure Infiltration Technique. Wear.

[15] Ureña A., Rams J., Escalera M.D., Sánchez M. (2005). Characterization of Interfacial Mechanical Properties in Carbon Fiber/Aluminium Matrix Composites by the Nanoindentation Technique. Compos. Sci. Technol..

[16] Tang Y., Deng Y., Zhang K., Liu L., Wu Y., Hu W. (2008). Improvement of Interface between Al and Short Carbon Fibers by α-Al2O3 Coatings Deposited by Sol-Gel Technology. Ceram. Int..

[17] Lee C.W., Kim I.H., Lee W., Ko S.H., Jang J.M., Lee T.W., Lim S.H., Park J.P., Kim J.D. (2010). Formation and Analysis of SiC Coating Layer on Carbon Short Fiber. Surf. Interface Anal..

[18] Sarapure S., Satish B.M., Girish B.M. (2018). Basawaraj Microstructure and Mechanical Behavior of Magnesium Alloy AZ91 Hybrid Composites. IOP Conf. Ser. Mater. Sci. Eng..

[19] Tian W., Qi L., Zhou J., Guan J. (2014). Effects of the Fiber Orientation and Fiber Aspect Ratio on the Tensile Strength of Csf/Mg Composites. Comput. Mater. Sci..

[20] Wang X.J., Hu X.S., Wu K., Deng K.K., Gan W.M., Wang C.Y., Zheng M.Y. (2008). Hot Deformation Behavior of SiCp/AZ91 Magnesium Matrix Composite Fabricated by Stir Casting. Mater. Sci. Eng. A.

[21] Ohno M., Mirkovic D., Schmid-Fetzer R. (2006). Liquidus and Solidus Temperatures of Mg-Rich Mg-Al-Mn-Zn Alloys. Acta Mater..

[22] Anilan Ajukumar K., AjithKumar K.K., Kunjayyappan Ravikumar K., Deva Rajan T.P., Subramonia Pillai U.T., Chandrasekhara Pai B. (2012). Fabrication and Characterization of Short Carbon Fiber Reinforced AZ91 Mg Alloy Composites. Mater. Sci. Forum.

[23] Afsharnaderi A., Malekan M., Emamy M., Rasizadeh Ghani J., Lotfpour M. (2019). Microstructure Evolution and Mechanical Properties of the AZ91 Magnesium Alloy with Sr and Ti Additions in the As-Cast and As-Aged Conditions. J. Mater. Eng. Perform..

[24] Liu J., Qi L.H., Guan J.T., Ma Y.Q., Zhou J.M. (2012). Compressive Behavior of C Sf/AZ91D Composites by Liquid-Solid Extrusion Directly Following Vacuum Infiltration Technique. Mater. Sci. Eng. A.

[25] Russell-Stevens M., Todd R., Papakyriacou M. (2005). The Effect of Thermal Cycling on the Properties of a Carbon Fibre Reinforced Magnesium Composite. Mater. Sci. Eng. A.

[26] Olszówka-Myalska A., Myalski J. (2015). Magnesium Alloy AZ31—Short Carbon Fiber Composite Obtained by Pressure Die Casting. Solid State Phenom..

[27] Fu S.Y., Lauke B., Mäder E., Yue C.Y., Hu X. (2000). Tensile Properties of Short-Glass-Fiber- and Short-Carbon-Fiber-Reinforced Polypropylene Composites. Compos. Part A Appl. Sci. Manuf..

[28] Kandemir S., Gavras S., Dieringa H. (2021). High Temperature Tensile, Compression and Creep Behavior of Recycled Short Carbon Fibre Reinforced AZ91 Magnesium Alloy Fabricated by a High Shearing Dispersion Technique. J. Magnes. Alloy.

[29] Daoud A. (2004). Wear Performance of 2014 Al Alloy Reinforced with Continuous Carbon Fibers Manufactured by Gas Pressure Infiltration. Mater. Lett..

